# Case Report: Multifocal plexiform neurofibromas presenting as a paratesticular “string-of-beads” mass in a 7-year-old boy with neurofibromatosis type 1

**DOI:** 10.3389/fped.2026.1900387

**Published:** 2026-07-15

**Authors:** Shuai Zhang, Yakun Xu, Jing Zhang, Lei Liu, Dianyong Liu

**Affiliations:** Department of Pediatric Urology, Dalian Women and Children's Medical Group, Dalian, China

**Keywords:** neurofibromatosis type 1, paratesticular, pediatric, plexiform neurofibroma, retroperitoneal tumor, scrotal mass, spermatic cord

## Abstract

**Background:**

Neurofibromatosis type 1 (NF1) is a common inherited tumor-predisposition syndrome with wide phenotypic variability. Plexiform neurofibromas (PNs) are characteristic NF1-associated peripheral nerve sheath tumors that may be extensive, infiltrative, and clinically silent. Genitourinary involvement is uncommon in children, and scrotal or paratesticular presentation with concomitant pelvic or retroperitoneal disease is rarely documented.

**Case presentation:**

A 7-year-old boy who met the revised clinical diagnostic criteria for NF1 presented with a 5-month history of a painless right scrotal “string-of-beads” mass. Ultrasonography showed bilaterally normal testes and multiple right inguinal extratesticular nodules continuous with an irregular paratesticular mass posterior and superior to the right testis, favoring a paratesticular/spermatic cord–adjacent process rather than a primary intratesticular tumor. Tumor markers were unremarkable. Staged CT disclosed additional multifocal lesions, including a right parietal subcutaneous lesion and a thoracic paravertebral lesion, together with substantial retroperitoneal and pelvic tumor burden partially encasing the iliac vessels without definite adjacent organ invasion. Testis-sparing scrotal exploration comprised incisional biopsy of the paratesticular lesion, excision of one superficial scrotal skin nodule, and excision of three local inguinoscrotal nodular specimens submitted as lymph-node-like tissue. Histopathology confirmed PN in the sampled paratesticular lesion and additional superficial nodular specimens; immunohistochemistry supported a benign peripheral nerve sheath tumor, with a Ki-67 labeling index of approximately 5%. Because the deep component was anatomically unresectable but not associated with pain, urinary obstruction, neurologic deficit, functional compromise, or definite adjacent organ invasion, structured surveillance was chosen rather than immediate MEK inhibitor therapy. At 6 months, the child remained clinically stable, but no interval MRI or CT had been performed.

**Conclusions:**

In a child with NF1 stigmata, an apparently superficial scrotal or paratesticular lesion may signal more extensive inguinal, pelvic, retroperitoneal, and paravertebral disease. Evaluation should extend beyond the scrotum, with MRI preferred when feasible. Management should be compartment-based: organ-preserving diagnostic surgery for accessible superficial disease, MRI-oriented surveillance for asymptomatic unresectable internal disease, and MEK inhibition for progressive, symptomatic, or function-threatening unresectable PN.

## 1 Introduction

Neurofibromatosis type 1 (NF1; MIM #162200) is an autosomal dominant tumor-predisposition syndrome caused by pathogenic variants in *NF1*, the gene encoding neurofibromin, a negative regulator of RAS/MAPK signaling. Approximately half of cases arise *de novo*, and clinical expression varies widely despite near-complete, age-dependent penetrance (1, 2). The earliest pediatric manifestations are usually pigmentary—café-au-lait macules and intertriginous freckling—whereas plexiform neurofibromas (PNs), optic pathway glioma, skeletal lesions, neurodevelopmental problems, and other systemic complications tend to emerge later (1–3).

PNs are clinically important because they typically arise early in life, involve multiple nerve fascicles, and may extend diffusely along tissue planes and neurovascular bundles. Although histologically benign, their clinical impact is often determined as much by anatomic extent and functional threat as by microscopic appearance. A subset may progress along the atypical neurofibromatous neoplasm of uncertain biologic potential (ANNUBP)–malignant peripheral nerve sheath tumor (MPNST) spectrum, which is increasingly assessed using integrated histopathologic and molecular criteria in diagnostically challenging or worrisome lesions (4, 5). Contemporary NF1 care therefore emphasizes early recognition, MRI-oriented extent mapping when feasible, longitudinal surveillance, selective surgery, and targeted therapy for carefully selected symptomatic, inoperable lesions (6–8).

Genitourinary involvement in pediatric NF1 is uncommon and is reported more often as lower urinary tract or pelvic disease than as isolated external genital disease. In a critical review of 79 pediatric cases, irritative lower urinary tract symptoms and an incidentally discovered abdominopelvic mass were common presentations, and a retrovesical mass with direct bladder involvement was a frequent imaging finding (9). Reported sequelae include urinary retention, obstructive uropathy, hydronephrosis, recurrent infection, and renal impairment (9).

Published reports nevertheless indicate that the pediatric genitourinary spectrum extends beyond bladder-predominant disease, encompassing external genital enlargement, clitoromegaly, penile involvement, scrotal or perineal masses, and mixed superficial-deep lesions that track from the inguinoscrotal region into the pelvis or retroperitoneum (10–15). Such cases matter because a superficially apparent genital finding may be localized, but it may also represent the distal manifestation of a larger internal plexiform process. Failure to recognize this possibility can lead to underestimation of tumor burden, incomplete staging, inappropriate local surgery, or delayed detection of organ-threatening complications (7, 9).

We report a 7-year-old boy with clinically diagnosed NF1 who presented with a painless right scrotal “string-of-beads” mass and was ultimately found to have multifocal PNs with extensive inguinal, pelvic, and retroperitoneal involvement. This case illustrates a practical diagnostic lesson: in a child with NF1 stigmata, an apparently superficial scrotal or paratesticular lesion should not be interpreted only as a local scrotal finding but may serve as a sentinel sign of more extensive internal disease, warranting whole-patient evaluation, MRI-based extent mapping when feasible, and compartment-based management (2, 7, 8).

## 2 Case description

### 2.1 Clinical history

A 7-year-old boy was referred for evaluation of a painless right scrotal mass that his parents had first noticed approximately 5 months earlier. The lesion initially appeared as a small nodule and then enlarged gradually. No previous diagnostic procedure or therapeutic intervention had been performed for this scrotal/paratesticular lesion before referral to our department. He had no history of trauma, scrotal pain, fever, hematuria, dysuria, urinary frequency, constipation, or other lower urinary tract symptoms. During the preceding 3 months, he had no weight loss but showed no interval weight gain, and his appetite was reportedly poor. Developmental milestones and school performance were age appropriate.

Neither parent had café-au-lait macules, neurofibromas, or other NF1-related features on clinical examination, and no first- or second-degree relative was known to have NF1 or another hereditary disorder. A timeline of the presentation, diagnostic work-up, treatment, and follow-up is provided in Table 1.

**Table 1:** Timeline of clinical events.

Time point	Event	Key findings	Management
5 mo before admission	Symptom onset	Small painless right scrotal nodule, gradually enlarging; no trauma, fever, pain, or urinary symptoms	Observation at home
3 mo before admission	Interval evolution	No weight loss but no weight gain; poor appetite	Medical evaluation eventually sought
Day 0–1	Clinical evaluation and ultrasonography	Firm, non-tender, beaded right hemiscrotal mass distinct from the normal right testis, extending proximally toward the inguinal region; left side normal; NF1 cutaneous stigmata present. Ultrasonography: both testes normal (right 1.5 × 1.2 × 0.9 cm; left 1.5 × 1.2 × 0.8 cm); CDFI: preserved intratesticular perfusion bilaterally; no abnormal intratesticular vascular signal; right inguinal hyperechoic nodular lesions (∼70 × 9 mm) continuous with an irregular extratesticular scrotal mass (∼29 × 12 mm)	Extratesticular or paratesticular process favored; imaging staging pursued
Day 1–2	Laboratory assessment and staging	Tumor markers not suggestive of germ cell tumor (ferritin 27.04 ng/mL, *β*-HCG <0.100 mIU/mL, AFP 0.67 IU/mL, CEA 1.89 ng/mL, CA125 27.12 U/mL); staged CT showed multifocal lesions — right parietal subcutaneous, thoracic paravertebral, and a substantial retroperitoneal/pelvic mass partially encasing the iliac vessels without definite adjacent organ invasion	Germ cell tumor considered unlikely, but other paratesticular or spermatic-cord lesions were not excluded; MDT review reclassified the presentation as multifocal disease
Day 3	Testis-sparing exploration with incisional biopsy	Cord-like plexiform paratesticular lesion; testis and epididymis preserved; one superficial scrotal skin nodule and three local inguinoscrotal nodular specimens submitted as lymph-node-like tissue were excised for pathological assessment	Organ-preserving diagnostic sampling
POD 1–4	Pathology, recovery, and discharge	Benign plexiform neurofibroma in the sampled paratesticular lesion and additional superficial nodular specimens; supportive immunohistochemistry; Ki-67 ∼5%; no malignant features; uneventful postoperative course	Radical resection and systemic therapy deferred; discharged on active surveillance with predefined escalation triggers
6-mo follow-up	Outpatient reassessment	Clinically stable on symptom review and physical examination; no scrotal progression; no new urinary, neurologic, or compressive symptoms; no interval MRI or CT completed	Surveillance continued; MRI-based follow-up planned to establish a longitudinal baseline

AFP, alpha-fetoprotein; β-HCG, beta-human chorionic gonadotropin; CA125, cancer antigen 125; CDFI, color Doppler flow imaging; CEA, carcinoembryonic antigen; CT, computed tomography; MDT, multidisciplinary team; mo, month(s); MRI, magnetic resonance imaging; NF1, neurofibromatosis type 1; POD, postoperative day.

### 2.2 Physical examination

Cutaneous examination revealed at least six café-au-lait macules measuring ≥5 mm over the trunk and extremities, together with intertriginous freckling involving the left axilla and both popliteal fossae. These pigmentary findings met two independent diagnostic criteria for NF1 under the revised consensus criteria (2). No discrete cutaneous or subcutaneous nodular neurofibromas were palpable. Ophthalmologic assessment showed no Lisch nodules or choroidal abnormalities.

**Figure 1:**
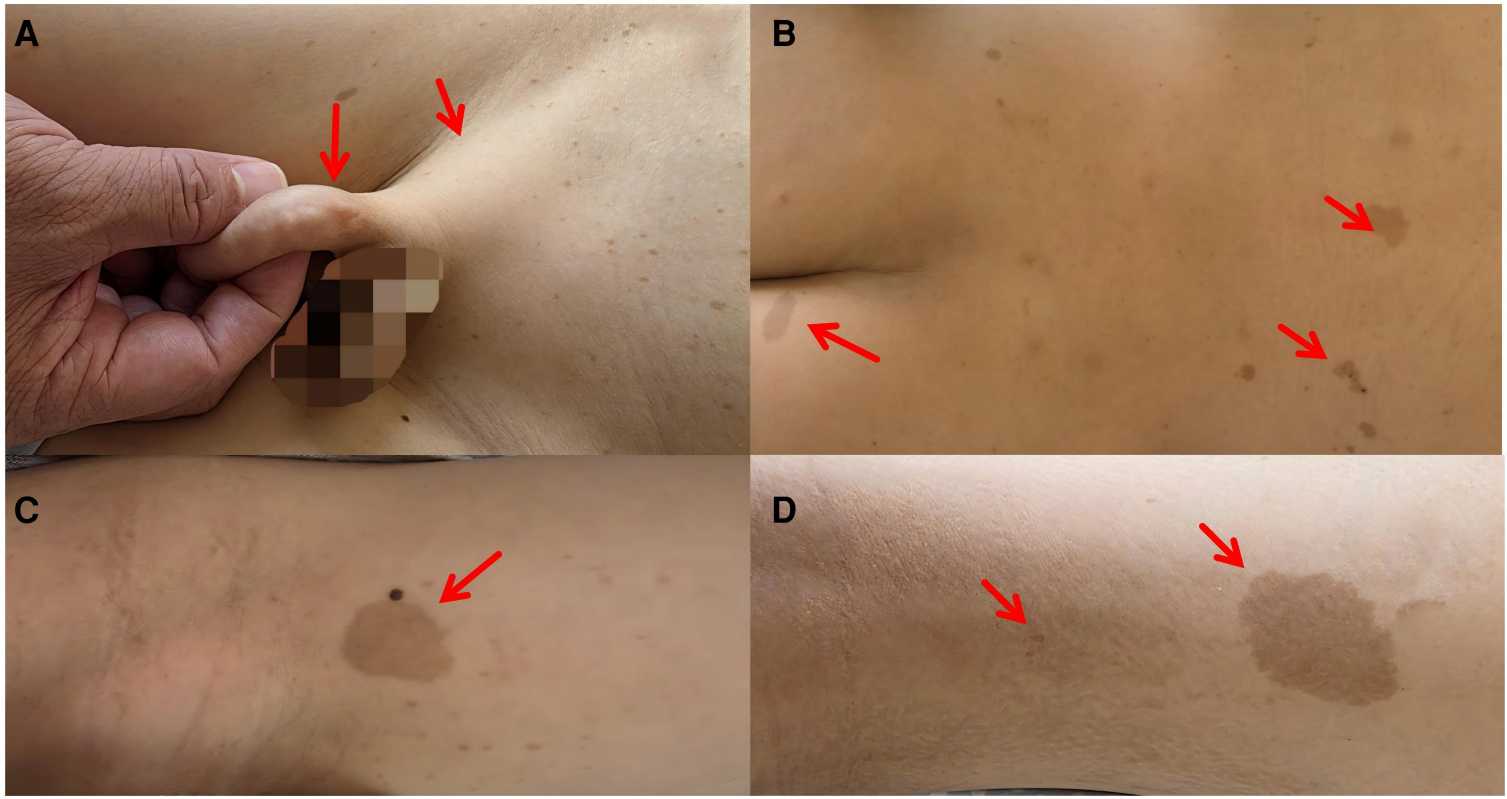
Clinical presentation and NF1-related cutaneous findings. **(A)** Masked clinical photograph showing a superficial beaded right inguinoscrotal lesion with proximal extension toward the right inguinal region, representing the clinically visible sentinel lesion; red arrows indicate the palpable beaded lesion and its proximal extension. **(B–D)** Representative café-au-lait macules in the trunk, proximal lower-limb, and axillary regions. The area shown in **(C)** also exhibited intertriginous freckling clinically, although this is only faintly appreciable in the photograph. The small dark pigmented lesion adjacent to the café-au-lait macule in **(C)** was regarded as an incidental benign-appearing melanocytic nevus. Red arrows indicate representative pigmentary findings.

Genitourinary examination showed enlargement of the right hemiscrotum. On careful palpation, the lesion appeared separate from the testis: the right testis was independently palpable, intact in contour, and of normal consistency, whereas a firm, non-tender, beaded mass was present within the right hemiscrotum, posterior and superior to the testis. The mass extended proximally in a cord-like fashion toward the right inguinal region, where an additional elongated mass was also palpable. In the context of the child's NF1 pigmentary features, this beaded/cord-like configuration raised clinical suspicion for a plexiform neurofibroma, although other pediatric paratesticular or spermatic-cord lesions remained in the differential diagnosis. The overlying skin was intact, without erythema, discoloration, or ulceration. The left testis and epididymis were unremarkable on palpation, and no mass was palpable in the left hemiscrotum or left inguinal region. There was no clinically evident inguinal hernia. Representative clinical findings, including the superficial right inguinoscrotal sentinel lesion and NF1-related cutaneous features, are shown in Figure 1.

Neurologic examination was normal, with intact cranial nerve function and no motor or sensory deficit. No scoliosis or kyphosis was noted. Cardiovascular, respiratory, and abdominal examinations were otherwise unremarkable.

### 2.3 Investigations

#### 2.3.1 Ultrasonography

Ultrasonography of the scrotum and inguinal region showed both testes to be morphologically normal and intrascrotal in position. According to the ultrasonography report, the right testis measured 1.5 ×  1.2 × 0.9 cm and the left testis measured 1.5 × 1.2 × 0.8 cm; both showed homogeneous echotexture without focal abnormality. Color Doppler imaging demonstrated preserved intratesticular perfusion bilaterally, with no abnormal intratesticular vascular signal.

The abnormal findings were clearly extratesticular. Along the right inguinal region, densely packed, mildly hyperechoic nodular foci were seen over an extent of approximately 70 × 9 mm, with relatively well-defined margins and scant internal vascularity. Inferiorly, this abnormality continued into the right scrotum, where an irregular hypoechoic mass posterior and superior to the right testis measured approximately 29 × 12 mm, with heterogeneous echotexture and sparse internal vascularity. Overall, ultrasonography showed multiple right inguinal extratesticular nodules in continuity with an irregular right scrotal extratesticular mass adjacent to the spermatic cord, with both testes spared. In the context of the child's NF1 pigmentary features and the beaded/cord-like configuration, plexiform neurofibroma was considered the leading diagnosis, although other pediatric paratesticular or spermatic-cord lesions remained in the differential diagnosis.

#### 2.3.2 Computed tomography

Staged computed tomography (CT) of the head, chest, abdomen, and pelvis revealed multifocal disease. Non-contrast head CT showed an approximately 18 × 4 mm subcutaneous soft-tissue lesion in the right parietal region. Chest CT showed a left paravertebral fusiform low-attenuation lesion alongside the T7–T10 thoracic spine, measuring approximately 20 × 10 mm in maximal axial dimensions. Contrast-enhanced abdominopelvic CT with multiplanar reconstruction showed a large low-attenuation retroperitoneal mass extending along the para-aortic/lumbar plexus region and tracking caudally into the pelvis; it measured approximately 7.2 cm in maximal craniocaudal extent, with axial dimensions of about 5.7 × 3.1 cm, and partially encased the iliac vessels without definite invasion of adjacent pelvic organs. Taken together, the imaging pattern favored multifocal NF1-associated peripheral nerve sheath tumors rather than an isolated scrotal lesion.

MRI is the preferred modality for mapping and longitudinal surveillance of NF1-associated plexiform neurofibroma in children because of its superior soft-tissue contrast and absence of ionizing radiation. In this case, CT was used only as the initial rapid staging modality because the patient presented with an unusual pediatric paratesticular/inguinoscrotal mass, there was concern for a possible malignant paratesticular or spermatic-cord tumor, and prompt cross-sectional assessment was needed to define the gross anatomic extent of disease. The child had limited tolerance for a prolonged MRI examination at presentation, and MRI under sedation or general anesthesia was not pursued as part of the initial rapid-staging workflow. We acknowledge that MRI, including sedation- or anesthesia-assisted MRI when necessary, would have been preferable for pediatric PN assessment and should be prioritized for subsequent evaluation. CT was not used to biologically characterize the lesions and cannot reliably differentiate benign plexiform neurofibroma from ANNUBP or MPNST.

#### 2.3.3 Laboratory tests

**Figure 2:**
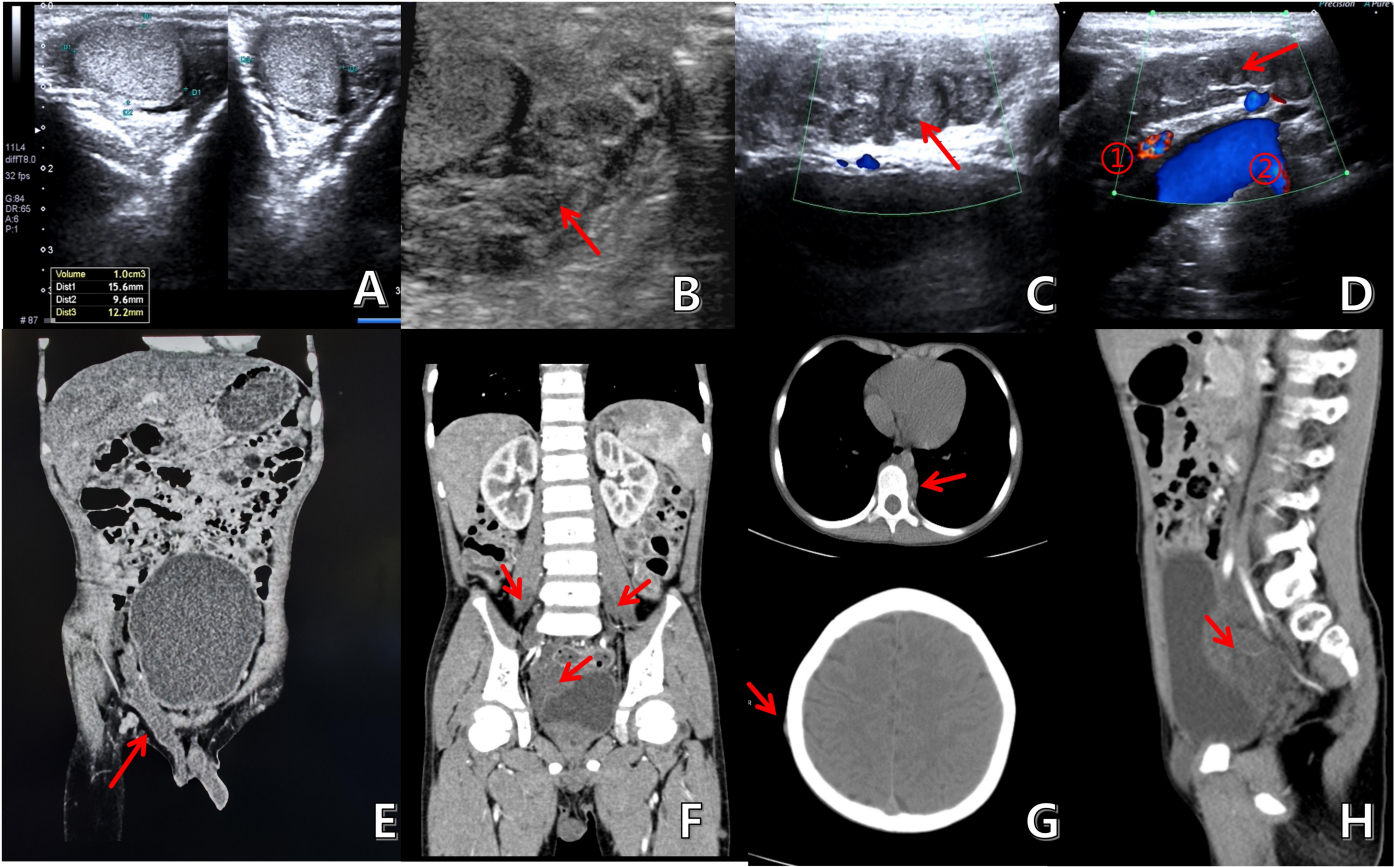
Ultrasonographic and CT findings of an extratesticular inguinoscrotal lesion with deep pelvic and retroperitoneal extension. **(A)** Scrotal ultrasonography showing the right testis with preserved contour and homogeneous echotexture, without focal intratesticular abnormality; the measured dimensions on the representative image were approximately 15.6 × 9.6 × 12.2 mm. **(B)** Irregular extratesticular lesion located posterior and superior to the right testis. **(C)** Beaded/nodular extratesticular lesions extending along the right inguinal course. **(D)** Color Doppler ultrasonography showing the inguinal lesion in close relationship to the iliac vessels, with ① indicating the iliac artery and ② indicating the iliac vein. **(E)** Coronal CT showing right inguinoscrotal extension contiguous with a large pelvic mass. **(F)** Coronal contrast-enhanced CT showing multiple irregular retroperitoneal and pelvic masses, including lesions adjacent to the iliac vessels. **(G)** Additional multifocal lesions on CT: the upper image shows a thoracic paravertebral lesion, and the lower image shows a right parietal scalp/subcutaneous lesion. **(H)** Sagittal CT showing a large pelvic mass posterior to the bladder with substantial craniocaudal extent. Red arrows indicate representative lesions or anatomic relationships in each panel.

Complete blood count, renal and liver function, serum electrolytes, and coagulation profile were within normal limits. Tumor marker assessment showed ferritin 27.04 ng/mL, β-human chorionic gonadotropin <0.100 mIU/mL, alpha-fetoprotein 0.67 IU/mL, carcinoembryonic antigen 1.89 ng/mL, and cancer antigen 125 27.12 U/mL. These values did not suggest a malignant germ cell tumor. However, normal tumor markers could not exclude other clinically important pediatric paratesticular or spermatic-cord lesions, including non-germ-cell malignancies. Representative ultrasonographic and CT findings—the normal right testis, the adjacent extratesticular inguinoscrotal lesion, contiguous inguinal extension, and the deep pelvic and retroperitoneal tumor burden—are shown in Figure 2.

### 2.4 Surgery, histopathology, and immunohistochemistry

The patient underwent testis-sparing scrotal exploration. Intraoperatively, a cord-like, beaded plexiform lesion was identified in the paratesticular soft tissue adjacent to the spermatic cord and in continuity with the inguinal course. The testis and epididymis were uninvolved and well perfused. Because the superficial lesion appeared to represent only the accessible portion of a larger proximal process, the operative strategy prioritized diagnostic confirmation and organ preservation rather than attempted definitive local excision.

Although plexiform neurofibroma was considered the leading diagnosis because of the child's clinical NF1 features and the beaded/cord-like appearance of the lesion, diagnostic sampling was performed because this was an unusual pediatric paratesticular presentation, MRI characterization was not available at initial staging, and other clinically important paratesticular or spermatic-cord lesions, including malignant tumors, remained in the differential diagnosis. An incisional biopsy with limited sampling of the paratesticular/spermatic cord–adjacent lesion was performed. In addition, one small superficial scrotal skin nodule and three small local inguinoscrotal nodular specimens, submitted intraoperatively as lymph-node-like tissue, were excised for pathological assessment.

Frozen section suggested a benign peripheral nerve sheath tumor with plexiform architecture in a myxoid stroma, without necrosis or significant cytologic atypia. Final histopathology confirmed plexiform neurofibroma. The lesion showed multinodular expansion of nerve fascicles by bland spindle cells in a myxoid background, without necrosis, significant cytologic atypia, or increased mitotic activity. A formal mitotic count was not reported. Immunohistochemistry supported the diagnosis, showing diffuse S-100 and SOX10 positivity, EMA-positive perineurial elements, focal NSE and GFAP positivity, and a Ki-67 labeling index of approximately 5%. H3K27me3, p16, p53, and CD34 were not evaluated because the sampled lesion lacked histological features suggestive of atypical neurofibromatous neoplasm of uncertain biologic potential or malignant peripheral nerve sheath tumor.

The excised superficial scrotal skin nodule and the three local inguinoscrotal nodular specimens submitted as lymph-node-like tissue were also diagnosed as plexiform neurofibroma. No malignant features were identified in any submitted specimen. In conjunction with the plexiform architecture, myxoid stroma, and absence of necrosis, cytologic atypia, or increased mitotic activity, these findings were consistent with benign plexiform neurofibroma under established histopathologic criteria and integrated diagnostic recommendations for NF1-associated peripheral nerve sheath tumors (4, 5, 16). In this context, the Ki-67 labeling index of approximately 5% did not, by itself, support atypical biology or overt malignant transformation.

**Figure 3:**
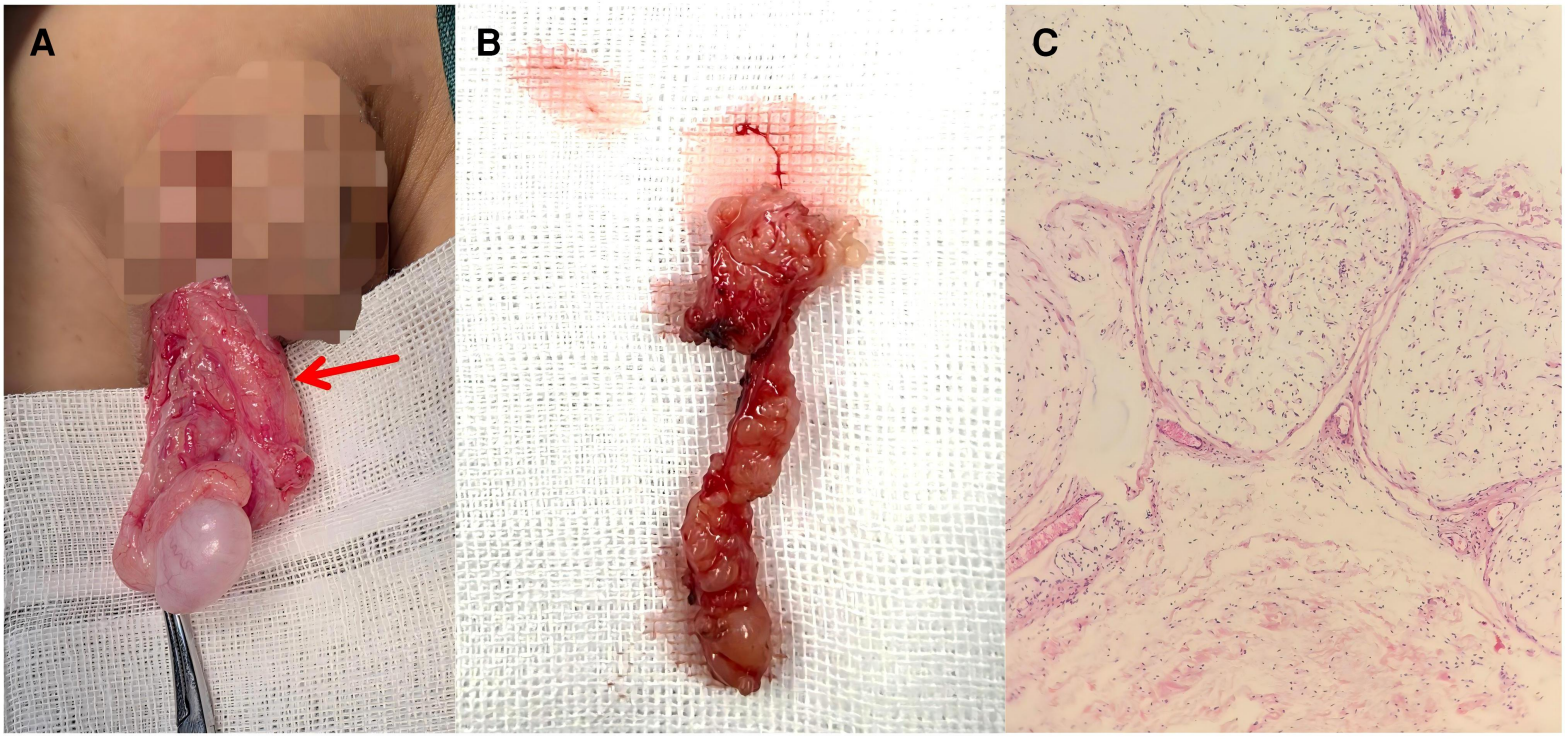
Intraoperative findings, gross specimen, and histopathologic confirmation of plexiform neurofibroma. **(A)** Masked intraoperative photograph showing the superficial elongated paratesticular/spermatic cord–adjacent lesion; the red arrow indicates the accessible superficial component selected for diagnostic sampling. **(B)** Gross specimen showing the beaded/plexiform appearance of the sampled superficial component. **(C)** Histopathology showing multinodular plexiform proliferation of bland spindle cells involving expanded nerve fascicles in a myxoid stroma, consistent with plexiform neurofibroma (hematoxylin and eosin staining, low-power view). Immunohistochemical findings, including epithelial membrane antigen (EMA) expression and the Ki-67 labeling index, are described in the main text. The additional superficial scrotal skin nodule and three local inguinoscrotal nodular specimens submitted as lymph-node-like tissue were also diagnosed as plexiform neurofibroma.

Because only the accessible superficial component was sampled, histological findings from these specimens could not directly establish the biological behavior of the deep pelvic and retroperitoneal tumor burden. Therefore, assessment of the deep component remains indirect and requires longitudinal MRI-based surveillance, preferably with volumetric assessment when feasible. If future MRI shows rapid growth, increasing heterogeneity, necrosis, new pain, neurologic deficit, urinary obstruction, or other concerning features, targeted reassessment, including targeted biopsy of the most suspicious region, would be considered. The intraoperative appearance of the superficial lesion, the gross specimen, and the supporting histopathology are shown in Figure 3.

### 2.5 Genetic testing

Germline *NF1* testing was discussed with the family but was not required to establish the clinical diagnosis or guide immediate management in this case. The child fulfilled the revised clinical diagnostic criteria for NF1 on the basis of characteristic pigmentary findings, and the diagnosis was further supported by pathologically confirmed plexiform neurofibroma. Therefore, molecular testing would mainly have served a confirmatory or research purpose and was not expected to predict disease course or alter the initial management strategy.

### 2.6 Treatment and follow-up

Given the extent of the retroperitoneal and pelvic tumor burden and its relationship to major neurovascular and visceral structures, the multidisciplinary team judged that complete resection of the deep component would carry substantial neurologic and visceral morbidity and would be unlikely to be curative. Because the superficial scrotal/paratesticular lesion appeared to represent the distal manifestation of more extensive proximal disease, management focused on diagnostic confirmation and testis preservation, followed by structured surveillance rather than attempted radical resection.

Postoperative recovery was uneventful. The deep pelvic and retroperitoneal component was anatomically unresectable but was not associated with pain, urinary obstruction, neurologic deficit, functional impairment, or other compressive symptoms at initial assessment. Although the initial CT defined the gross anatomic extent of disease, it was not used to biologically characterize the deep component. After multidisciplinary review and discussion with the family, immediate MEK inhibitor therapy was deferred, while preserving it as an escalation option if the lesion became progressive, symptomatic, function-threatening, or associated with paravertebral/spinal risk.

Structured surveillance was adopted with predefined triggers for escalation, including radiographic progression, rapid or asymmetric enlargement, new or worsening pain, functional impairment, urinary obstruction, neurologic deficit, increasing imaging heterogeneity, necrosis, invasive behavior, or other compressive symptoms. MRI-based follow-up, preferably with volumetric assessment when feasible, was planned to establish a longitudinal baseline for the pelvic, retroperitoneal, and paravertebral components and to monitor tumor growth, morphology, warning signs of malignant transformation, and other NF1-related complications.

At the 6-month outpatient follow-up, the child remained clinically stable, with a well-healed incision, no progression of the superficial scrotal findings on physical examination, and no new pain, urinary symptoms, neurologic deficit, or compressive symptoms. No interval MRI or CT had been completed at that time; therefore, radiological stability of the deep pelvic and retroperitoneal component could not be confirmed.

## 3 Discussion

In this patient, a palpable paratesticular “string-of-beads” mass shifted the clinical problem from local scrotal triage to whole-patient evaluation for multifocal NF1-associated PN. The principal contribution of this report is therefore practical rather than anecdotal: in a child with clear NF1 stigmata, a beaded or cord-like inguinoscrotal lesion should prompt early consideration of PN and should not be managed as an isolated scrotal finding. The superficial inguinoscrotal sentinel lesion led to extent mapping that reframed an apparently localized extratesticular lesion as the distal manifestation of a broader plexiform process. This reframing mattered clinically because management, surveillance intensity, imaging strategy, and family counseling all change once internal pelvic and retroperitoneal disease is recognized.

Against the existing pediatric literature, our case occupies an instructive middle ground. Most scrotal masses in children are benign and are initially triaged by ultrasonography, whereas neurogenic tumors of the scrotal or paratesticular region are rare (17). Lesions reported in children as “scrotal neurofibroma” are frequently isolated intrascrotal or paratesticular neurofibromas, without clear evidence of generalized NF1 or major internal extension (18–22). By contrast, pediatric NF1 with genitourinary involvement more often presents through bladder or prostatic urethral disease, lower urinary tract obstruction, hydronephrosis, or macrogenitalia (10–14). Our patient bridged these two patterns: the presentation was a superficially apparent scrotal/paratesticular lesion, yet further evaluation uncovered extensive deep pelvic and retroperitoneal disease. This supports an iceberg effect, in which the visible genital finding is the most accessible end of a much larger internal plexiform tumor burden. Key published pediatric NF1 cases with genitourinary involvement are summarized in Supplementary Table S1.

Three practical points deserve emphasis. First, the pigmentary findings were diagnostically decisive rather than incidental. In a child with no family history, multiple café-au-lait macules and intertriginous freckling are sufficient to establish the clinical diagnosis of NF1 under the revised consensus criteria when other diagnostic conditions are excluded, and the diagnosis in this patient was further supported by pathologically confirmed PN (2). Germline *NF1* testing may have confirmatory or research value, but it was not required to establish the clinical diagnosis or guide immediate management in this case.

Second, imaging should not stop once an extratesticular lesion has been identified. Ultrasonography correctly localized the lesion to the paratesticular/spermatic cord–adjacent region and suggested proximal continuity, but formal extent mapping was needed to determine whether the lesion represented a localized scrotal process or part of more extensive internal disease. MRI is the preferred modality for pediatric NF1-associated PN assessment and longitudinal surveillance because of its superior soft-tissue contrast and absence of ionizing radiation (7, 8). In this patient, CT was used only as the initial rapid staging modality to define gross anatomic extent when there was concern for an unusual paratesticular or spermatic-cord tumor and possible occult internal disease. CT did not biologically characterize the lesions and cannot reliably differentiate benign PN from ANNUBP or MPNST. Subsequent evaluation should therefore prioritize MRI-based surveillance, preferably with volumetric assessment when feasible.

Third, tissue confirmation should be interpreted in the context of sampling limitations. Although the beaded/cord-like appearance in a child with clear NF1 stigmata made PN the leading diagnosis, this was an unusual pediatric paratesticular presentation, MRI characterization was unavailable at initial staging, and other clinically important paratesticular or spermatic-cord lesions remained in the differential diagnosis. Conservative, testis-sparing diagnostic sampling helped confirm PN and exclude other malignancies in the sampled superficial component, while avoiding premature radical surgery. However, because histopathology sampled only the accessible superficial component, the biological behavior of the deep pelvic and retroperitoneal tumor burden cannot be directly inferred from these specimens and depends on longitudinal imaging-based surveillance.

The differential diagnosis of a pediatric extratesticular or inguinoscrotal mass is broad and should be approached systematically. Common benign conditions include inguinal hernia or hydrocele, varicocele-like venous lesions, inflammatory or reactive nodules, lymphatic malformation, and lipomatous lesions. Clinically important neoplastic considerations include paratesticular rhabdomyosarcoma and other spermatic-cord or paratesticular tumors. In the present case, several findings argued against a purely local or nonsyndromic lesion: the mass was clinically separable from the testis, had a beaded or cord-like configuration, extended proximally toward the inguinal region, and occurred in a child with diagnostic NF1 pigmentary stigmata. Ultrasonography further localized the lesion to the extratesticular/paratesticular region and suggested proximal continuity, whereas further staging disclosed multifocal internal disease involving the inguinal, pelvic, retroperitoneal, and paravertebral compartments. These findings supported NF1-associated PN rather than an isolated scrotal lesion.

Histologic benignity alone does not capture clinical risk in NF1-associated PN; what matters clinically is whether the tumor is enlarging, threatening function, or showing warning signs of transformation. Current guidance and integrated diagnostic recommendations highlight rapid or asymmetric enlargement, new or worsening pain, hardening, progressive neurologic deficit, marked imaging heterogeneity, necrosis, invasive behavior, and increased ^1^⁸F-FDG uptake as features that raise concern for ANNUBP or MPNST and may warrant targeted biopsy with sampling adequate for histologic and, when indicated, molecular assessment (4, 5, 7, 8). In our patient, the sampled superficial component showed benign plexiform morphology without atypia, increased mitotic activity, or necrosis; the Ki-67 labeling index of approximately 5% did not, by itself, support atypical biology; and no malignant features were identified in any submitted specimen. However, the deep component was not directly sampled, and its biological interpretation remains indirect. MRI-based follow-up, preferably with MR-volumetry when feasible, is therefore important to establish a longitudinal baseline and detect further growth, particularly because age is associated with PN growth and younger children may show faster growth rates (23).

Management could not be reduced to a binary decision about resection. The superficial scrotal/paratesticular component was accessible to organ-preserving diagnostic surgery with limited sampling, whereas the deep pelvic and retroperitoneal component was extensive, closely related to the iliac vessels and other major structures, and not amenable to safe definitive excision. This mixed-resectability pattern supports compartment-based management, in which anatomy, symptoms, growth behavior, and functional risk—rather than visibility alone—govern the intensity of intervention.

MEK inhibition has transformed the treatment of symptomatic, inoperable NF1-associated PNs. The SPRINT phase 2 trial showed durable tumor shrinkage and functional benefit with selumetinib in children with inoperable PN (24), and consensus statements recommend selumetinib as first-line medical therapy for symptomatic lesions that cannot be fully resected without unacceptable morbidity (7, 8). The ReNeu phase IIb trial subsequently established mirdametinib as another option for adults and children with symptomatic, inoperable NF1-associated PN, with reported objective responses and improvements in pain and health-related quality of life (25). In addition, published data suggest that spinal or paravertebral neurofibroma burden may respond to selumetinib in patients with NF1-associated PN (26). In our patient, the deep pelvic, retroperitoneal, and paravertebral components were anatomically inoperable, yet unresectability alone did not mandate immediate systemic therapy. Because the child had no pain, neurologic deficit, urinary obstruction, functional impairment, or other compressive symptoms at the time of assessment, structured surveillance after multidisciplinary review represented a proportionate interim strategy, while preserving MEK inhibition as an escalation option if MRI follow-up demonstrates progressive growth, increasing paravertebral/spinal risk, functional threat, or other clinically significant progression.

Multidisciplinary review combined anatomic extent, pathology, current functional status, imaging limitations, family counseling, and the timing of possible escalation into a single management decision, with input from pediatric urology, radiology, pathology, dermatology, ophthalmology, and tumor-focused specialists (6). Treatment planning should therefore start from disease extent and functional risk, not from the most visible lesion.

Several limitations should be acknowledged. This is a single case, so its conclusions are inferential rather than definitive. Germline molecular testing was not performed; however, the child fulfilled the revised clinical diagnostic criteria for NF1, and molecular confirmation was not required for clinical diagnosis or immediate management. Somatic molecular profiling of the surgical specimen was likewise not undertaken, as the sampled lesion lacked atypical features that would trigger an integrated diagnostic work-up (5). The initial internal extent was characterized mainly by CT rather than MRI; CT was useful for gross anatomic staging but cannot replace MRI for pediatric PN characterization or longitudinal surveillance and cannot reliably distinguish benign PN from ANNUBP or MPNST. In addition, the deep pelvic and retroperitoneal component was not directly sampled, so the benign histology of the superficial component cannot exclude spatial heterogeneity within the deeper tumor burden. Finally, follow-up remains short, and no interval MRI or CT had been completed at the 6-month clinical follow-up; therefore, radiological stability of the deep component could not be confirmed. These limitations constrain the scope of the report without negating its central message: in a child with NF1 stigmata, an apparently local extratesticular scrotal lesion can expose a much larger, clinically occult internal tumor burden that requires MRI-oriented surveillance and compartment-based management.

## 4 Conclusion

Acknowledging the limits of single-case evidence, this report broadens the documented pediatric spectrum of NF1-associated genitourinary plexiform neurofibroma and illustrates a clinically useful principle: in a child with NF1 stigmata, an unusual extratesticular scrotal lesion should prompt syndromic thinking rather than purely local interpretation. Once PN is suspected, evaluation should extend beyond the scrotum to define occult pelvic, retroperitoneal, and paravertebral disease, with MRI preferred for extent mapping and longitudinal surveillance when feasible. Management should be compartment-based and risk-stratified, guided by disease extent, growth behavior, symptoms, and functional threat rather than by the visibility of the superficial lesion alone. Organ-preserving diagnostic surgery may be appropriate for accessible superficial disease, whereas unresectable internal disease requires structured MRI-oriented surveillance and timely escalation, including consideration of MEK inhibition, if progression, symptoms, or function-threatening features emerge.

## Patient/guardian perspective

Because the patient was a minor, the perspective was provided by his legal guardian. The family initially sought medical evaluation because they were concerned about the gradual enlargement of a painless right scrotal mass. After imaging showed that the clinically visible inguinoscrotal lesion was associated with deeper pelvic and retroperitoneal disease, the parents were anxious about the extent of the tumor burden and the potential need for major surgery or systemic therapy. After multidisciplinary counseling, they understood that the deep component was not suitable for safe complete resection at that time and that immediate systemic treatment was not mandatory in the absence of pain, functional impairment, urinary obstruction, neurologic deficit, or other concerning clinical features. They agreed with the plan of structured surveillance, with MRI-based follow-up and predefined triggers for treatment escalation.

## Data Availability

The original contributions presented in the study are included in the article/Supplementary Material, further inquiries can be directed to the corresponding author.

## Supplementary material

Supplementary Table S1 Pediatric NF1 with genitourinary involvement (≤18 years), stratified by predominant anatomic site.
