# Supplementary material for: Case Report: Multifocal plexiform neurofibromas presenting as a paratesticular “string-of-beads” mass in a 7-year-old boy with neurofibromatosis type 1
Source: Front Pediatr. 2026 Jul 15;14:1900387. doi: 10.3389/fped.2026.1900387 (PMC13416410; doi:10.3389/fped.2026.1900387)
Supplement: Supplementary Table S1 — Pediatric NF1 with genitourinary involvement (≤18 years), stratified by predominant anatomic site. [file Table1.docx]

**Supplementary Material**

**Supplementary Table S1.** Pediatric NF1 with genitourinary involvement (≤18 years), stratified by predominant anatomic site.

| **Reference** | **Age/Sex** | **NF1 basis/status** | **Presentation** | **Predominant site**  **(s) involved** | **Pathology** | **Management** | **Outcome/follow-up** |
| --- | --- | --- | --- | --- | --- | --- | --- |
| Jepson (1975) (1) | 5/M | Clinical NF1 reported | Asymptomatic periurethral scrotal–perineal mass | Periurethral scrotal–perineal plexiform lesion | Plexiform neurofibroma reported | Excision with urethroplasty | NR† |
| Rink and Mitchell (1983) (2) | 2 children; ages NR† | Clinical NF1; pediatric genitourinary neurofibromatosis series | Variable genitourinary symptoms with or without external genital findings | Bladder, ureter, pelvis, and external genitalia (variable by case) | Neurofibroma/plexiform neurofibroma reported | Case-based surgical management according to site and severity | As reported |
| Ogawa and Watanabe (1986) (3) | Boy; age NR† | Clinical NF1 | Enlarged penis and scrotum with urinary retention | Pelvic mass involving bladder outlet/prostatic urethra with obstructive uropathy | Plexiform neurofibroma reported | Urinary diversion after failure of catheter drainage | Hydronephrosis managed by diversion |
| Pascual-Castroviejo et al. (2008) (4) | 4 children; 3F/1M; ages NR† | Clinical NF1 (familial or sporadic, reported) | External genital enlargement and/or pain | Clitoris, labium majus, penis; pelvic extension adjacent to the bladder in some cases | Plexiform neurofibroma reported in most cases | Biopsy, excision, or reconstructive planning according to lesion site | As reported |
| Dündar et al. (2010) (5) | 6/M | Clinical NF1 | Macrogenitalia with chronic renal failure | Bladder and prostatic urethra with lower urinary tract obstruction | Genitourinary neurofibromatosis/neurofibroma reported | Management for obstructive uropathy and renal failure | As reported |
| Yesodharan et al. (2017) (6) | 5/F | Clinical/familial NF1 reported | Clitoromegaly | Clitoris | Plexiform neurofibroma | Surgical excision with neurovascular preservation | Follow-up not clearly reported |
| Rabley et al. (2019) (7) | Female child; age NR† | Genetically confirmed NF1 | Painful clitoromegaly | Clitoris | Plexiform neurofibroma | Excision/clitoroplasty | NR† |
| An et al. (2025) (8) | 17/M | Clinical NF1 reported | Enlarged scrotum and penis affecting function/quality of life | External genitalia (penis/scrotum); deep extent not clearly documented | Neurofibroma/plexiform features reported | Testis-preserving debulking and reconstruction | As reported |
| El Mahi et al. (2025) (9) | 10; child with male genital involvement† | Clinical NF1 | Scrotal swelling with progressive penile enlargement | Intrapelvic extension involving the bladder and spermatic cords | Neurofibroma reported | Conservative follow-up | Stable/asymptomatic on follow-up |
| Present case | 7/M | Clinical NF1 by revised criteria; multifocal PN; genetic testing not completed | Painless right scrotal “string-of-beads” mass | Paratesticular/spermatic cord–adjacent lesion with inguinal, pelvic, retroperitoneal, and thoracic paravertebral involvement; additional right parietal subcutaneous lesion | Plexiform neurofibroma in the sampled paratesticular lesion, superficial scrotal skin nodule, and three local inguinoscrotal nodular specimens submitted as lymph-node-like tissue; Ki-67 ∼5%; no malignant features | Testis-sparing scrotal exploration with incisional biopsy and limited superficial sampling; deep component not resected; structured MRI-oriented surveillance | Clinically stable at 6 months based on symptom review and physical examination; no interval MRI or CT completed; MEK inhibitor therapy deferred but retained as an escalation option |

***Abbreviations:*** *F, female; M, male; NF1, neurofibromatosis type 1; NR, not reported (or not verifiable from accessible source); PN, plexiform neurofibroma.*

***Footnotes: † Indicates that the field could not be verified from accessible primary-source abstract or full-text material at the time of manuscript preparation. No demographic or follow-up details were imputed when unavailable.***

***Table note: This table summarizes published pediatric NF1 cases with genitourinary involvement (≤18 years) and stratifies them by predominant anatomic pattern. The present case is included for direct comparison. Fields that could not be confirmed from accessible primary-source material are conservatively marked NR.***

**References cited in Supplementary Table S1**

1. Jepson PM. Von Recklinghausen’s disease presenting as scrotal tumor. Urology. (1975) 5:270–4. doi: 10.1016/0090-4295(75)90030-8

2. Rink RC, Mitchell ME. Genitourinary neurofibromatosis in childhood. J Urol. (1983) 130:1176–9. doi: 10.1016/S0022-5347(17)51743-7

3. Ogawa A, Watanabe K. Genitourinary neurofibromatosis in a child presenting with an enlarged penis and scrotum. J Urol. (1986) 135:755–7. doi: 10.1016/S0022-5347(17)45841-1

4. Pascual-Castroviejo I, López-Pereira P, Martínez-Bermejo A, López-Gutiérrez JC, Cisternino M, Lobato RD. Neurofibromatosis type 1 with external genitalia involvement: presentation of 4 patients. J Pediatr Surg. (2008) 43:1998–2003. doi: 10.1016/j.jpedsurg.2008.01.074

5. Dündar BN, Oktem F, Armağan A, Dündar NO, Bircan S, Yesildag A. Chronic renal failure and macrogenitalia associated with genitourinary neurofibromatosis. Pediatr Nephrol. (2010) 25:353–6. doi: 10.1007/s00467-009-1308-5

6. Yesodharan D, Sudarsanan B, Jojo A, Abraham M, Bhavani N, Mathews H, et al. Plexiform neurofibroma of clitoris. J Pediatr Genet. (2017) 6:244–6. doi: 10.1055/s-0037-1602789

7. Rabley A, Bayne CE, Shenoy A, DeMarco RT. Genital neurofibromatosis presenting as painful clitoromegaly. Urology. (2019) 133:219–21. doi: 10.1016/j.urology.2019.07.016

8. An J, Wang J, Bao Z. Surgical excision of genitourinary neurofibromatosis complicated by enlarged scrotum and penis: a case report. Urol Case Rep. (2025) 61:103085. doi: 10.1016/j.eucr.2025.103085

9. El Mahi N, Mojahid A, Siouri H, Ziani H, Nasri S, Kamaoui I, et al. Rare uro-genital manifestations of von Recklinghausen disease: scrotal, penile, and intrapelvic involvement with bladder and spermatic cord extension: a case report. Radiol Case Rep. (2025) 20:3116–9. doi: 10.1016/j.radcr.2025.03.008
